# The role of IL-17 in the pathogenesis and treatment of glioblastoma—an update on the state of the art and future perspectives

**DOI:** 10.1007/s12032-024-02434-1

**Published:** 2024-06-25

**Authors:** Dariusz Łaszczych, Aleksandra Czernicka, Karol Gostomczyk, Łukasz Szylberg, Jędrzej Borowczak

**Affiliations:** 1 Department of Obstetrics, Gynaecology and Oncology, Collegium Medicum, Nicolaus Copernicus University in Bydgoszcz, Ujejskiego 75 street, 85-168 Bydgoszcz, Poland; 2 Department of Tumor Pathology and Pathomorphology, Oncology Centre – Prof. Franciszek Łukaszczyk Memorial Hospital, dr Izabeli Romanowskiej 2 street, 85-796 Bydgoszcz, Poland; 3 Department of Clinical Oncology, Oncology Centre – Prof. Franciszek Łukaszczyk Memorial Hospital, dr Izabeli Romanowskiej 2 street, 85-796 Bydgoszcz, Poland

**Keywords:** Glioblastoma, Interleukin-17, Th17, Tumorigenesis, Tumor microenvironment

## Abstract

Glioblastoma (GBM) is the most common malignant brain tumor, which, despite significant progress made in the last years in the field of neuro-oncology, remains an incurable disease. GBM has a poor prognosis with a median survival of 12–15 months, and its aggressive clinical course is related to rapid growth, extensive infiltration of adjacent tissues, resistance to chemotherapy, radiotherapy and immunotherapy, and frequent relapse. Currently, several molecular biomarkers are used in clinical practice to predict patient prognosis and response to treatment. However, due to the overall unsatisfactory efficacy of standard multimodal treatment and the remaining poor prognosis, there is an urgent need for new biomarkers and therapeutic strategies for GBM. Recent evidence suggests that GBM tumorigenesis is associated with crosstalk between cancer, immune and stromal cells mediated by various cytokines. One of the key factors involved in this process appears to be interleukin-17 (IL-17), a pro-inflammatory cytokine that is significantly upregulated in the serum and tissue of GBM patients. IL-17 plays a key role in tumorigenesis, angiogenesis, and recurrence of GBM by activating pro-oncogenic signaling pathways and promoting cell survival, proliferation, and invasion. IL-17 facilitates the immunomodulation of the tumor microenvironment by promoting immune cells infiltration and cytokine secretion. In this article we review the latest scientific reports to provide an update on the role of IL-17 role in tumorigenesis, tumor microenvironment, diagnosis, prognosis, and treatment of GBM.

## Introduction

Glioblastoma (GBM) is the most common primary malignant neoplasm of the central nervous system (CNS), reaching an incidence rate of 3.23 per 100,000 in the USA [1]. GBM has an aggressive clinical course, with a median survival of 12–15 months [2]. Despite optimal therapy, only 6.8% of patients survive 5 years from diagnosis [1]. GBM has a high recurrence rate with half of the patients experiencing disease progression within 7.4 months [3]. Short progression-free survival and frequent relapses are due to high biological malignancy of the tumor, defined by cell infiltration far beyond the tumor boundaries seen by neuroimaging or macroscopically during tumor resection.

Recently, glioblastoma stem cells (GCS) have arisen as the main source of glioblastoma self-renewal ability, which predetermines its recurrence after resection or radio/chemotherapy. GSCs are located in specific regions of the tumor microenvironment (TME). While there is no clear consensus regarding the subdivision of TME compartments, TME is generally divided into perivascular, perinecrotic/hypoxic, and immune niches [4]. Most stem cells are located in hypoxic and perinecrotic niches of GBM, which are characterized by the imbalance between rapid cancer cells growth and sluggish blood flow [5]. This specific arrangement of GSCs has several clinical implications [5]. Firstly, insufficient blood supply impairs the penetration of chemotherapeutics and facilitates GSCs survival, playing a key role in GBM relapse [5]. Secondly, hypoxic conditions in these two niches promote various adaptive mechanisms within stem cells that contribute to the development of tumor radioresistance [6].

The crosstalk between tumor and stromal cells is regulated by a complicated cytokine network driving local inflammation and immunosuppression [7]. Recently, the family of interleukins has gained attention as the key driver of disease progression. Interleukins modulate the development and differentiation of various immune cells, affecting the non-specific immune response by recruiting neutrophils or activating macrophages [8].

Among them, interleukin 17 (IL-17), a proinflammatory cytokine secreted primarily by T helper 17 (Th17) lymphocytes, emerged as a major regulator of glioblastoma cells proliferation and migration [9]. There are two main mechanisms in which IL-17 drives tumor cell development. Through direct binding with its receptor, IL-17 activates numerous transcription factors, protein kinases, metalloproteinases, and anti-apoptotic proteins, directly stimulating tumor cells [10–12]. Indirectly, IL-17 induces the immunosuppressive TME and inhibits the anticancer immune response [13, 14]. IL-17 upregulates the expression of transcription factors such as B-lymphoma Mo-MLV insertion region 1 (BMI1) in GBM cells [15]. BMI1 maintains the self-renewal capacity of GSC, and its overexpression stimulates their proliferation [16]. Furthermore, increased plasma level of IL-17 is associated with a higher risk of glioma [17]. Th17-associated cytokines including IL-1β, IL-17, and IL-23 are significantly elevated in GBM patients [18], and the increased presence of IL-17^+^ cells was associated with a worse prognosis in GBM patients [19]. Interestingly, some studies showed opposite results. IL-17 expression significantly correlated with longer PFS and lower mortality rates, and was an independent predictor of good prognosis in patients with GBM [20]. The inconclusive results of past studies indicate that more research is needed to fully understand the significance of IL-17 in tumorigenesis and prognosis in GBM. A better understanding of the effect of IL-17 on GBM progression and the application of targeted therapy against IL-17 signaling may constitute a promising therapeutic approach in GBM.

In recent years, our understanding of the role of IL-17 in GBM tumorigenesis has been greatly enhanced by several high-quality studies. Remarkably, many years have passed since the implications of IL-17 and glioblastoma were summarized [21]. Therefore, in this state-of-the-art review, we discuss the role of IL-17 in the pathogenesis, diagnosis, prognosis, and treatment of GBM. In our discussion, we include the role of IL-17 in various crucial processes during tumorigenesis, including cellular aspects (proliferation, migration, and cell survival), cancer stem cells, angiogenesis, and the interaction network between cells in the tumor microenvironment. We also review the current knowledge on the role of IL-17 in the diagnosis, prognosis and treatment of GBM. With growing evidence that the IL-17 signaling pathway is a key factor in GBM pathogenesis, targeted therapy against IL-17 signaling is emerging as a promising therapeutic modality.

## The IL-17 family and IL-17^+^ cells in health and disease

IL-17A (commonly named IL-17) modulates immune response by playing a key role in the induction of innate response [22]. While Th17 lymphocytes constitute the main source of IL-17, it can also be produced by CD8^+^ cytotoxic lymphocytes, macrophages, γδ T cells, mast cells, and natural killer cells (NK) [23]. Th17, neutrophils, γδ T cells, and resident glial cells (astrocytes and microglia) are the main sources of IL-17 in the CNS [24]. The differentiation of naive T lymphocytes into Th17 lymphocytes depends on interleukin 6 (IL-6) and transforming growth factor β (TGF-β), which induce the expression of the transcription factor retinoid-related orphan receptor Ɣ (RORγt) [25]. RORγt stimulates the expression of IL-23 receptors on the surface of Th17 lymphocytes. Interleukin 23 (IL-23) is produced by antigen-presenting cells like dendritic cells (DC), macrophages, and microglia. IL-23 upon binding with its receptor on the surface of Th17 cells induces secretion of IL-17 [26].

To date, the role of IL-17 signaling is best understood in the pathogenesis of colorectal cancer (CRC) [27]. While IL-17 promotes cancer development by affecting the TME, the underlying mechanisms are likely universal to most tumors. Up-regulation of IL-17 starts at an early stage of CRC development and IL-17 level positively correlates with the severity of dysplasia in adenomas [28]. Several studies showed that IL-17 plays a key role in CRC metastasis and prognosis [29]. IL-17 has also been implicated in the oncogenesis of pancreatic, ovarian, gastric, hepatocellular, prostate, skin, and breast cancer [30]. Targeting IL-17 may enhance the efficacy of immune checkpoint inhibitors and anti-angiogenic agents in colorectal cancer [31]. It seems that combined therapy of anti-IL-17 agents together with standard chemotherapy, immunotherapy, or radiotherapy is a promising novel therapeutic opportunity in cancer treatment. However, to evaluate the safety and efficacy of that approach more research is required.

## The role of IL-17 in tumorigenesis of glioblastoma

**Fig. 1:**
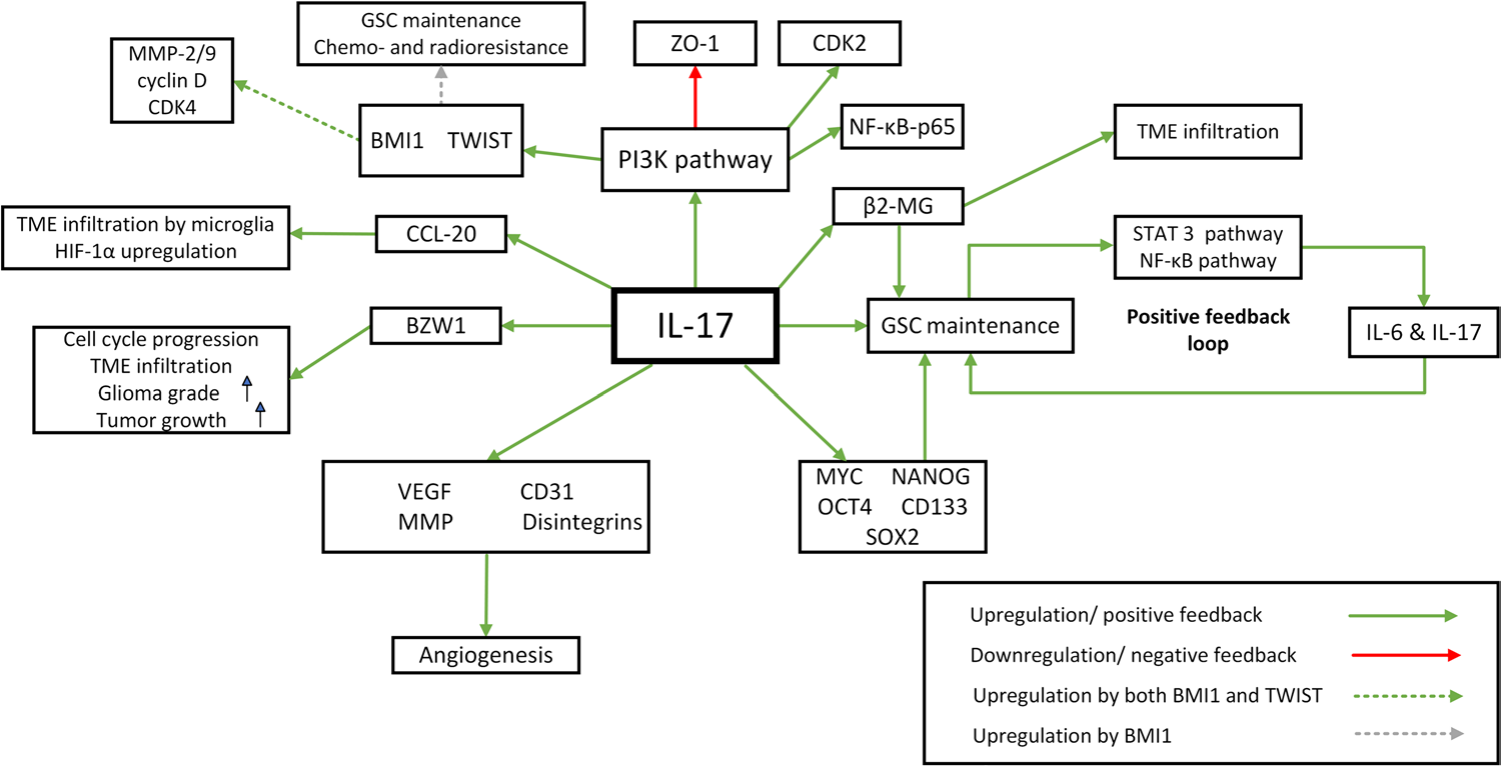
The role of IL-17 in GBM tumorigenesis. IL-17 binds with its receptor expressed on the surface of glioblastoma cells and activates the PI3K pathway, upregulating BMI1, TWIST, CDK2, and NF-kB-p65 signaling. BMI1 and Twist are key pro-oncogenic transcription factors that enhance the expression of MMP-2/9, cyclin D, and cyclin-dependent kinase 4 (CDK4). BMI1 maintains GSC and drives their chemo- and radioresistance. MMPs remodel the extracellular matrix and facilitate cancer cell migration. Cyclin D, CDK4, and CDK2 promote cell cycle progression and cancer cell proliferation. IL-17 upregulates β2-MG expression and promotes immune cell migration to TME. β2-MG enables GSC viability and self-renewal. Pro-GSC activity of IL-17 activates STAT3 and NF-kB pathways in a positive feedback loop mediated by IL-6 and IL-17. IL-17 upregulates glioblastoma stem cell markers, such as MYC, NANOG, OCT4, CD133, and SOX2, promoting GSC self-renewal. IL-17 drives angiogenesis in tumor tissue by upregulating VEGF, CD31, MMP, and disintegrins. IL17-induced CCL-20 promotes microglia infiltration and facilitates cancer cell survival in stressful conditions such as hypoxia. IL-17 may also upregulate the BZW1 oncogene, promoting cell cycle progression and increasing tumor aggressiveness. Notable, BZW1 expression correlates with glioma WHO grade. The PI3K pathway downregulates tight junction protein ZO-1, possibly increasing cancer cell migration and invasion

Carcinogenesis is a complex process that involves multidirectional interactions between cancer, immune, and stromal cells in the TME. Pro-inflammatory cytokines promote tumorigenesis, including proliferation, migration, angiogenesis, and escape from immunosurveillance, while IL-17 and Th17-promoting cytokines may be key factors mediating the crosstalk between cancer and stromal cells [32]. Recent studies showed that IL-6, IL-17, IL-23, and TGF-β are overexpressed in GBM tissue [15, 33, 34]. IL-17 promotes early-stage growth of GBM, elucidating its effects via several mechanisms (Fig. 1) [35]. In this section, we will explore the potential mechanisms by which IL-17 is involved in GBM tumorigenesis. This includes cellular aspects such as proliferation, cell survival, and migration. We also discuss the link between IL-17 and glioblastoma stem cells.

### The effect of IL-17 on PI3K/AKT pathway

The phosphatidylinositol-3-kinase (PI3K)/AKT signaling pathway is involved in cell growth, survival, proliferation, migration, and apoptosis. IL-17 enhances PI3K/AKT signaling in GBM cells, upregulating BMI1 and Twist transcription factors and leading to the overexpression of matrix metalloproteinases 2/9 (MMP-2/9) and cyclin D [15]. BMI1 is highly expressed in mesenchymal glioblastoma, a highly migratory tumor subtype. BMI1 promotes the ability of GSCs to self-renew and drives tumor chemo- and radioresistance [16]. MMPs degrade and remodel the extracellular matrix (ECM), allowing GBM to invade adjacent tissue [36]. IL-17-induced cyclin-dependent kinase 2 (CDK2)-cyclin D complex promotes cell cycle progression and GBM proliferation [37]. In addition, IL-17 downregulates the expression of the cell–cell tight junction protein zonula occludens-1 (ZO-1), which may reduce cell adhesion and promote GBM motility [15]. However, some authors reported that depleting ZO-1 can reduce cell mobility by disrupting actin network formation [38]. IL-17 receptors were significantly expressed on the surface of two glioblastoma cell lines. Furthermore, IL-17 stimulated phosphorylation of the transcription factor NF-κB-p65 and promoted GBM tumorigenesis via the PI3K/AKT pathway in a mouse model. Inhibition or knockdown of the PI3K pathway suppressed IL-17-induced GBM growth [9].

Results suggest that IL-17 may drive key processes during glioblastoma tumorigenesis including proliferation, migration, and therapy resistance through PI3K/AKT pathway. Notable, the PI3K/AKT axis promotes GBM progression, angiogenesis, metastasis, stemness, and resistance to standard chemotherapy including temozolomide [39]. Since IL-17 induces the PI3K/AKT pathway, it seems that targeting IL-17 and resulting down-regulation of the downstream PI3K/AKT axis may enhance chemotherapy efficiency and reduce the risk of relapse. In addition, combined therapy consisting of anti-IL-17 and PI3K inhibitors may exert even more significant anti-GBM effects. Therefore, we expect more research investigating this potential therapeutic approach in the near future.

### IL-17 contributes to glioblastoma stem cells maintenance

Inflammatory cytokines may play an important role in GBM progression by interacting with GSCs and TME. Nearly 15% of GBM cells express IL-17R and 75% of IL-17^+^ tumor cells co-express GSC markers [40]. IL-17 induces proliferation and self-renewal of GSC, which secrete significant amounts of pro-inflammatory cytokines and chemoattractants such as IL-8 and monocyte chemoattractant protein-1 (MCP-1). IL-17-induced cytokine secretion by GSC is associated with signal transducer and activator of transcription 3 (STAT3) and NF-κB pathways [40]. STAT3 and NF-κB pathways are activated in a positive feedback loop by GSC-derived IL-6 and IL-17. It seems that IL-17 promotes GSC maintenance through modulation of ongoing inflammation in the TME mediated by STAT3 and NF-κB signaling [40]. Notable, STAT3 is crucial for GSC maintenance and proliferation [41]. Alternatively, IL-17 may regulate GSC maintenance through interaction with the immune-related molecule β2-microglobulin (β2-MG). Overexpression of IL-17 in GBM cells increased the expression of β2-MG, which may be a critical factor in GSC proliferation, tumorigenicity, and self-renewal [35, 42]. Recently, it has been demonstrated that GBM cells transfected with IL-17 cDNA upregulated the stem cell markers. Inhibition of IL-17 significantly decreased the expression of stemness markers and the self-renewal ability of cancer cells, inhibiting tumor growth in vivo [34].

There is growing evidence that IL-17 may have a significant role in the maintenance of GSC through the promotion of inflammation in TME. It seems that effective anti-GBM treatment may be achieved by targeting glioblastoma stem cells. Therefore, novel therapies against GSC may be cutting-edge approaches to the treatment of GBM. More research is expected to investigate the link between IL-17 and GSC and how targeting IL-17 may affect the stemness properties of GBM.

### IL-17 and BZW1 oncogene

Basic leucine zipper and W2 domains protein 1 (BZW1) is a key regulator of transcriptional control at the G1/S transition during the cell cycle [43]. High BZW1 expression is associated with poor prognosis in pancreatic cancer, and BZW1 expression positively correlates with infiltration of the tumor microenvironment (TME) by CD8^+^ T cells, macrophages, and neutrophils [44]. BZW1 expression is elevated in glioma tissue and increases with glioma WHO grade, reaching the highest levels in GBM. In addition, BZW1 overexpression is associated with faster GBM growth in vivo and increased TME infiltration with CD4^+^, CD8^+^, and DCs [45]. Higher expression of BZW1 in GBM cells correlates with increased activity of the IL-17 signaling pathway, suggesting a potential link between BZW1 and IL-17 in GBM tumorigenesis [45].

To our knowledge, this is the first study to demonstrate the relationship between BZW1 and IL-17 in any neoplasm, not just GBM. It remains unclear whether IL-17 stimulation causes BZW1 upregulation in GBM cells or if BZW1 overexpression results in secondary activation of the IL-17 signaling pathway. Uncovering the connection between BZW1 and IL-17 will be crucial to advance our understanding of GBM development and progression.

### IL-17 promotes early-stage growth of GBM

GBM cells transfected with human IL-17 cDNA showed increased proliferation potential for the first 32 days in a murine xenograft model. However, on days 35 and 39, there was no difference in tumor size between transfected and control groups. This suggests that IL-17 may promote the early stages of GBM growth [35]. IL-17 transfection increased the expression of inflammatory and immune response molecules, such as chemokine C–C motif ligand 20 (CCL20) and β2-MG [35]. CCL20 promotes the infiltration and migration of microglial cells into GBM and favors cancer survival in a hypoxic environment, which is likely to occur during rapid early growth, by upregulating hypoxia-inducible factor 1-alpha (HIF-1alpha) [46, 47]. β2-MG expression positively correlated with infiltration of GBM TME by B cells, macrophages, neutrophils, and DC. High TME infiltration is associated with shorter survival, while β2-MG overexpression appears to be an unfavorable prognostic biomarker in GBM. The authors suggest that β2-MG may mediate immunosuppressive TME and resistance to immunotherapy [48].

It seems that the pro-oncogenic effect of IL-17, especially in the early stages, may be related to its pro-inflammatory and pro-survival activities. In addition, IL-17 may facilitate the early progression of GBM by attenuating host anti-tumor immunity through the upregulation of β2-MG, resulting in high immune cell infiltration of the TME. Since IL-17 appears to be an important factor in the early stage of GBM, targeting this cytokine may prevent rapid tumor growth and improve patient prognosis.

## IL23-IL17 *axis* and Th17-related cytokines in the GBM pathogenesis

IL-17 is involved in complex crosstalk with other cytokines and immune cells. An excessive and uncontrolled immune response mediated by IL-17 can lead to persistent inflammation. The entire IL-17 family is known to be involved in the pathogenesis of neuroinflammatory autoimmune diseases [49].

**Fig. 2:**
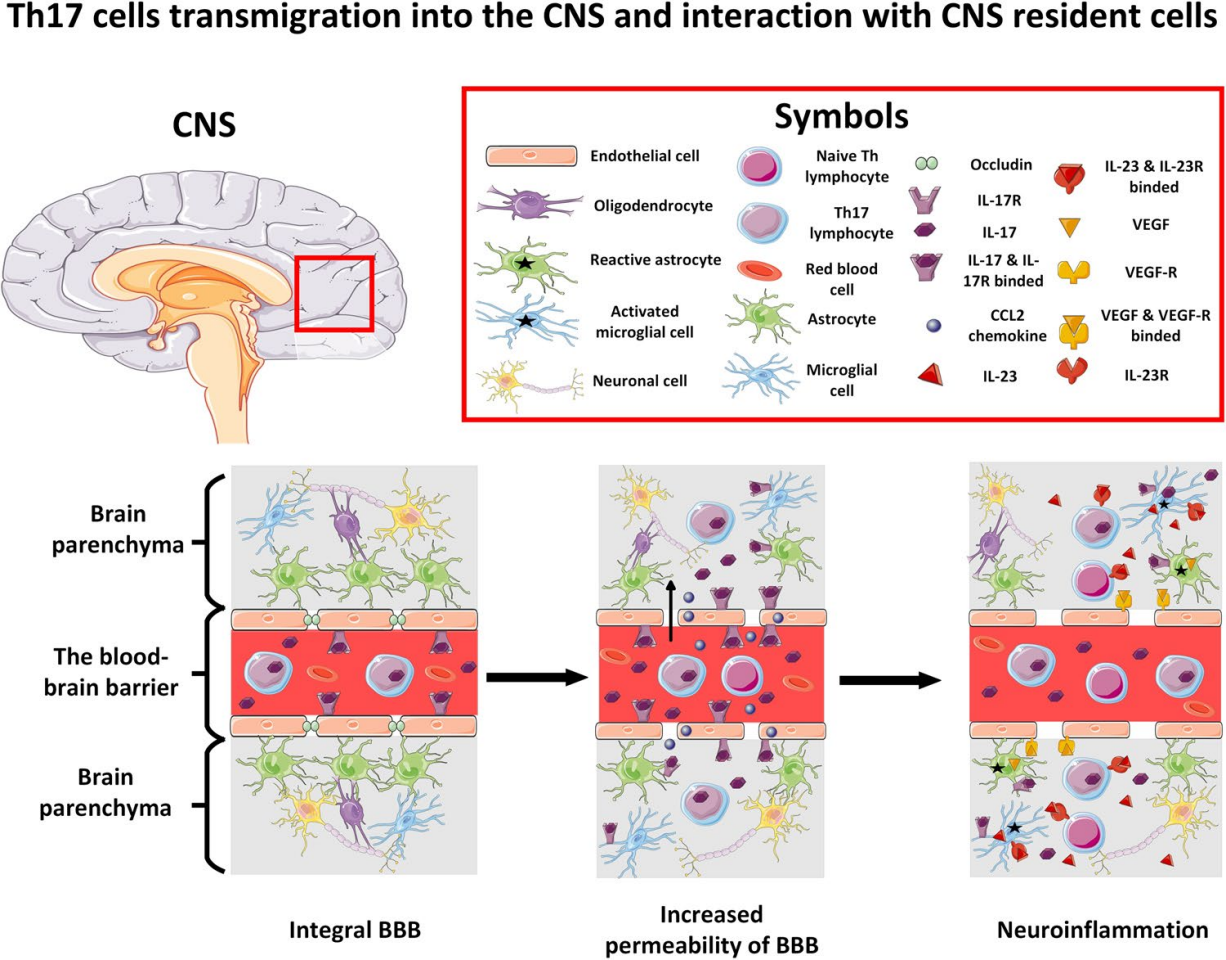
The proposed mechanisms of Th17 cell transmigration and their role in the initiation of neuroinflammation during GBM tumorigenesis. Th17 can access the CNS through several routes, but the main gateway is the microvasculature (venular route). IL-17 secreted by Th17 cells binds to its receptor on the endothelial surface, downregulates the expression of the tight junction protein occludin, and promotes endothelial cell contraction. It increases the permeability of the blood–brain barrier (BBB) and facilitates the migration of Th17 cells, naive T lymphocytes and other immune cells into the CNS. Within the CNS, IL-17 stimulates endothelial cells to secrete the chemokine CCL2, which promotes BBB leakage. In addition, IL-17 binds to its receptor on the surface of resident CNS cells such as astrocytes and microglial cells. Activation of resident CNS cells promotes a neuroinflammatory cascade associated with further BBB disruption and differentiation of naive T cells. IL-23, secreted by various antigen-presenting cells (not shown), can bind to its receptor on the microglial cell, resulting in the production of IL-17. In addition, activated microglial cells promote Th17 cell differentiation and maturation in autocrine signaling through IL-23 secretion. IL-17 stimulation upregulates VEGF expression in astrocytes. VEGF binds with its receptors on endothelial cells and increases BBB leakage and angiogenesis and promotes immune cell infiltration into the brain parenchyma. This figure was created using Servier Medical Art templates, licensed under a creative commons attribution 3.0 Unported License; https://smart.servier.com

Transmigration of Th17 into the CNS appears critical during GBM tumorigenesis. Th17 cells, as a source of tumor-promoting IL-17, facilitate GBM growth; however, little is known about how Th17 migrates within the brain parenchyma in GBM (Fig. 2). Potential mechanisms may be similar to those seen during ongoing neuroinflammation and extrapolated to the GBM model [50]. Th17 cells enter the CNS by several routes. The main gateway is a venular route through microvessels. Th17 can also migrate directly from the blood into the cerebrospinal fluid (CSF) via the choroidal route. IL-17 upregulates the expression of CCL20 on choroidal epithelial cells and promotes the entry of Th17 cells into the subarachnoid space [50]. IL-17 can disrupt the integrity of the blood–brain barrier (BBB) through downregulation of occludin and activation of the endothelial contractile machinery [51]. Transmigrated Th17 cells, by secreting IL-17, further stimulate the release of the chemokine CCL2 from endothelial cells, resulting in BBB disruption [52]. IL-17 binds to IL-17R, which is expressed on the surface of microglia and astrocytes [53]. Stimulated cells activate a neuroinflammatory cascade, causing further disruption of the BBB and differentiation of effector T cells [54]. Microglial cells produce IL-17 when stimulated by IL-1β and IL-23 stimulation. In addition, activated microglia secrete IL-23, which promotes Th17 cell differentiation and enhances IL-17 secretion in autocrine signaling [55]. IL-17 induces reactive astrogliosis, a change in the biochemical and physiological characteristics of astrocytes in response to ongoing pathology. Reactive astrocytes upregulate the expression of vascular endothelial growth factor (VEGF), which results in increased permeability of the blood–brain barrier (BBB), angiogenesis, and increased infiltration of immune cells into the brain parenchyma [56]. The crosstalk between reactive astrocytes and microglia leads to the release of TGF-β and IL-10, attenuating host anti-tumor immune response [57]. Furthermore, the presence of reactive astrocytes is associated with a poor prognosis and a reduced response to immunotherapy in GBM [58]. IL-17 also modulates the immunological properties of TME and attenuates the antitumor immune response.

Th17 cells are present in 80% of GBM tissue samples. However, it is unclear whether Th17 cells migrate from the peripheral blood across the BBB and into the TME or whether naive T cells differentiate into Th17 cells in situ [33]. Conversely, plasma concentrations of IL-17 and IL-23 are significantly elevated in GBM patients compared to healthy volunteers, and IL-17 transcript levels are decreased in GBM tissue compared to normal brain tissue [18]. The population of Th17 cells in peripheral blood is significantly higher in patients with high-grade glioma (HGG), including GBM, compared to low-grade glioma (LGG). Contrary to previous studies, patients with HGG and GBM had significantly increased levels of Th17-related cytokines in both plasma concentration and tissue mRNA transcript compared to LGG and healthy controls [34]. Cells within the TME may secrete Th17-related cytokines, as mRNA transcript levels were increased in GBM tissue.

Recent studies implicate alternative sources of Th17-related cytokines in the GBM milieu. Glioma-activated myeloid cells secrete Th17-promoting cytokines, including IL-1β, TGF-β, IL-6, and IL-23, and have a major impact on the induction of intratumoral Th17 cells in TME [59]. Myeloid-derived TGF-β increases the frequency of the immunosuppressive phenotype of Th17 (high IL-10 secretion) in the TME [59].

IL-6, one of the main cytokines involved in Th17 differentiation, can be secreted by resident microglial cells, GBM-associated endothelial cells in the vascular niche, and glioma cells [60–63]. IL-6 secretion from cancer cells increased after treatment with temozolomide [64]. Similarly, chemotherapy-induced changes in the cytokine profile of the TME including IL-17 and TGFβ downregulation and IL-23 upregulation [65]. GSC, a specific subpopulation of GBM cells, can secrete IL-23. This finding suggests that GSCs may be an alternative source of IL-23 in the TME [66].

**Fig. 3:**
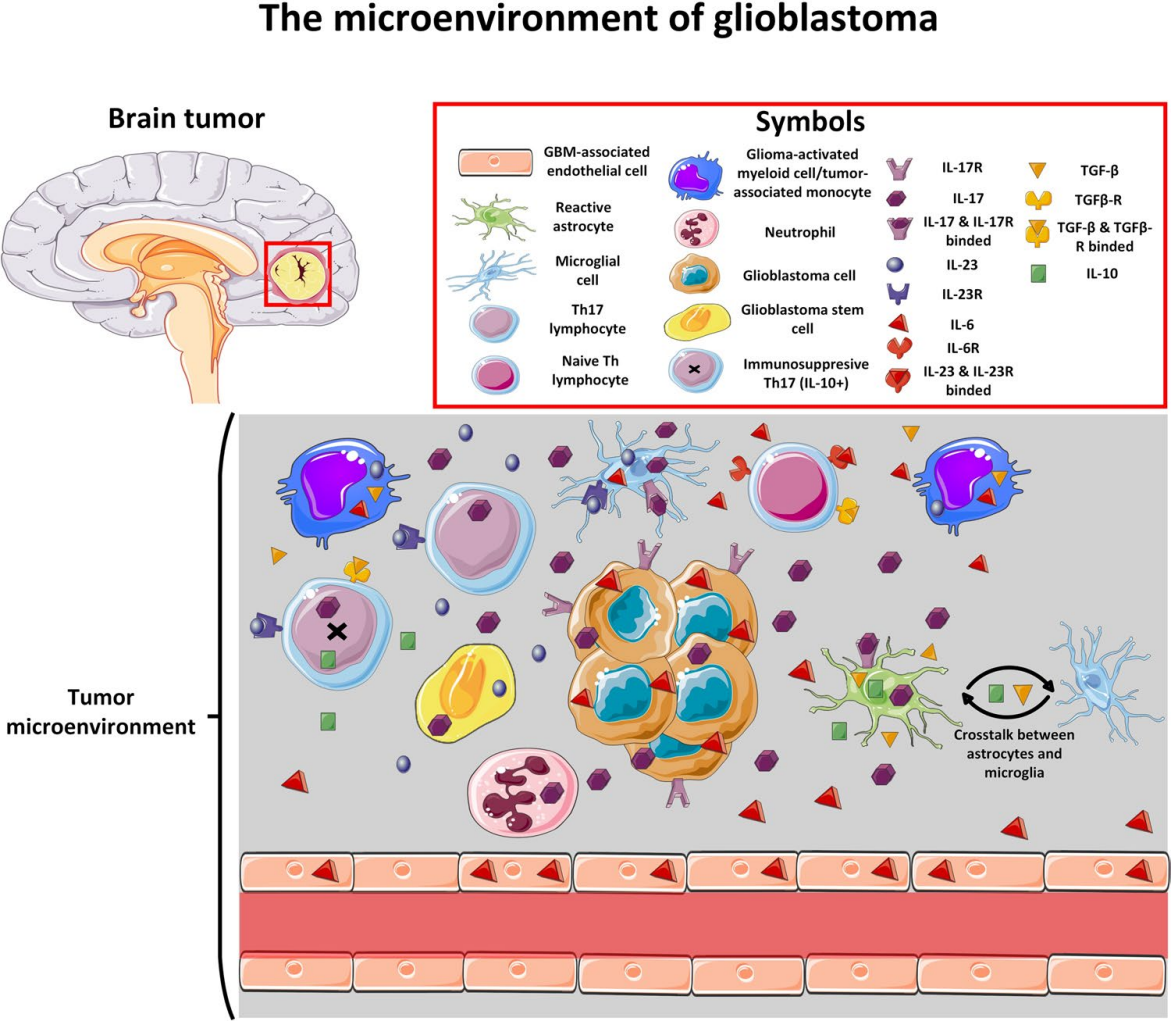
The tumor microenvironment (TME) is a specific microsystem composed of cancer cells, immune cells, and stromal cells together with extracellular matrix, blood vessels, and signaling molecules. Cells within the TME are interconnected through multidirectional interactions that promote tumor growth, invasion, immune surveillance evasion, and treatment resistance. In the case of GBM, Th17 cells can migrate from the peripheral blood across the BBB into the CNS or originate from naive T cells within the CNS. Key Th17-promoting cytokines such as TGF-β, IL-6 and IL-23 can be secreted by various cells present in the TME, including microglia (IL-6, IL-23), reactive astrocytes (TGF-β), GBM-associated endothelial cells (IL-6), glioma-activated myeloid cells/tumor-associated monocytes (IL-6, TGF-β, IL-23), glioblastoma cells (IL-6), and glioblastoma stem cells (IL-23). IL-17 can be secreted by Th17 cells, glioblastoma cells, glioblastoma stem cells, neutrophils, microglial cells, and reactive astrocytes. IL-17 promotes cancer cell survival, tumor growth, invasion of adjacent tissues, cancer stem cell self-renewal, and resistance to standard therapies. Due to its highly immunosuppressive microenvironment, GBM is considered an immunologically cold tumor, which has contributed to the failure of immunotherapy in GBM. IL-10, a key anti-inflammatory cytokine, is one of the main drivers of the immunosuppressive TME in GBM. It originates from immunosuppressive Th17 cells (stimulated by TGF-β secreted by myeloid cells) and resident CNS cells via specific crosstalk between astrocytes and microglia. This figure was created using Servier Medical Art templates, licensed under a Creative Commons Attribution 3.0 Unported License; https://smart.servier.com

In summary, the complexity of cellular interconnections suggests in addition to the classical IL23-IL17 axis described in the pathogenesis of various diseases, we can distinguish specific modifications of this pathway in GBM (Fig. 3). Several alternative sources of Th17-related cytokines have been described in the CNS and the TME of GBM. Specific crosstalk between cancer cells, microglia, reactive astrocytes, and immune cells seems to be crucial during GBM tumorigenesis. A better understanding of the interactions between these cells and the role of the IL23-IL17 axis in the development and maintenance of glioblastoma may provide insight into possible immunotherapeutic strategies in the treatment of this disease.

## IL-17 in angiogenesis and GBM pathogenesis

Angiogenesis plays a critical role in the growth and progression of GBM. IL-17 creates a favorable environment for angiogenesis by maintaining chronic inflammation [67]. It leads to the release of matrix metalloproteinases and disintegrins that degrade the ECM surrounding blood vessels [68]. Such a change creates a space for new vessels to form. Both immune cells and growth factors attracted to the site of inflammation also contribute to neoangiogenesis [69]. Angiogenesis stimulated by IL-17 and inflammation promotes the recruitment of immune cells, which can release more pro-inflammatory cytokines, including IL-17. Such a positive feedback loop sustains the inflammatory and angiogenic processes within the TME [70]. However, the process is a complex and evolving research field, and additional factors and interactions may require further investigation.

IL-17 may accelerate glioblastoma growth by upregulating angiogenesis-associated molecules, including VEGF and CD31 [35]. Upregulation of VEGF increases the vascularity of the TME and facilitates the migration of cancer cells [71]. Furthermore, IL-17 regulates glucose metabolism, angiogenesis, and adipogenesis in tumor tissue through increasing VEGF and HIF-1α expression, inducing glioblastoma growth in vivo [72]. In addition, IL-17^+^ areas of GBM are enriched in well-developed and neoangiogenic blood vessels, whereas GBM patients lacking IL-17^+^ cells have a reduced number of blood vessels. Patients with high levels of Th17 and VEGF had worse clinical outcomes than those with low levels of Th17 and VEGF [19].

IL-17 may promote vascularization in glioblastoma tissue by promoting inflammation and upregulating pro-angiogenic factors. VEGF plays a key role in angiogenesis and may be a potential prognostic biomarker in GBM [73]. Several anti-angiogenic agents targeting VEGF or VEGF receptors (VEGF-R) have been investigated as potential adjunctive treatments for recurrent GBM. To date, bevacizumab is the only FDA-approved anti-VEGF agent for recurrent GBM [73]. Of note, concomitant use with other anticancer agents may potentially optimize and enhance the anti-GBM effect of bevacizumab. Ibrahim et al. proposed that combined treatment with anti-IL-17 and anti-VEGF may increase the efficacy of therapy in metastatic colorectal cancer. However, to date, there are no preclinical or clinical studies investigating the significance of such an approach [74]. The association between IL-17 and VEGF suggests that combined therapy targeting both molecules may be a promising strategy in the treatment of GBM. Therefore, we expect further research in this area.

## IL-17 as a diagnostic marker in GBM

Elevated levels of IL-17 may indicate the presence of ongoing inflammation, which is characteristic of GBM [75]. Monitoring IL-17 levels can provide insight into tumor aggressiveness and the state of the immune response [76]. Assessment of IL-17 levels may aid in the diagnosis of CNS tumors. Patients with HGG have significantly increased levels of IL-17 in both plasma and mRNA transcript in tumor tissue compared to LGG [34]. In addition, serum IL-17 levels were significantly elevated in GBM compared to schwannoma, meningioma, and healthy volunteers [77].

The combination of thalidomide with or without temozolomide and radiotherapy decreased the serum concentration of IL-17 in GBM patients, suggesting that IL-17 could be used as a marker for GBM recurrence. However, further research is needed to investigate the potential role of IL-17 monitoring in the early detection of recurrence [78].

Unfortunately, the use of IL-17 as a diagnostic marker is limited by its low specificity. IL-17 is involved in several inflammatory and autoimmune diseases, and its elevated levels indicate inflammation in general, not just GBM [79]. Moreover, GBM is a complex and heterogeneous disease—its development and progression involve numerous molecular and cellular steps, and a single inflammatory marker such as IL-17 is unlikely to capture the complexity of the disease. Any potential application of IL-17 will require rigorous clinical and experimental validation. Large-scale studies in diverse patient populations are needed to determine the sensitivity, specificity, and predictive value of IL-17 in the diagnosis of GBM. Recent evidence suggests that the measurement of IL-17 in CSF has the potential to increase sensitivity and specificity in the diagnosis of GBM. CSF IL-17 and IL-10 are potential diagnostic biomarkers in patients with primary CNS lymphoma with sensitivity and specificity of 70% and 96%, respectively [80]. Since the CSF level of IL-17 is rather not affected by its extracranial production, the use of CSF instead of peripheral blood may increase the sensitivity and specificity of measurement. However, CSF collection is a more invasive procedure and carries the risk of serious adverse events. Therefore, the efficacy and safety of this method should be evaluated in future clinical trials.

## IL-17 as a prognostic marker in GBM

The prognostic value of IL-17 is the subject of active research and is not yet fully understood. IL-17 may also exert anti-tumor function by enhancing NK cells and cytotoxic T lymphocytes (CTLs) [81]. Despite its proinflammatory role in the TME, IL-17 is not only involved in immune activation but under certain conditions can also induce immunosuppression and reduce the anti-tumor response, allowing the tumor to evade immune surveillance and continue to grow. As a proangiogenic factor, IL-17 contributes to the aggressive course of the tumor, which worsens patient prognosis [81]. In two retrospective studies including a total of 263 patients treated with a standard radiochemotherapy protocol, the increased number of IL-17^+^ tumor-infiltrating cells in the TME was shown to be an independent factor for poor OS [19, 82]. Notably, IL-17 expression (assessed at both protein and mRNA levels) increased progressively with increasing WHO clinical classification of glioma (with the highest expression in WHO grade IV GBM). Since IL-17 expression positively correlates with the WHO grade of glioma tissue, it appears that IL-17 may be a potentially adverse prognostic biomarker in GBM patients [15]. On the contrary, IL-17 is associated with an increased 2-year survival rate, PFS, and a more favorable prognosis [20, 83].

In addition, baseline and endpoint levels of IL-17 may be a prognostic factor in GBM patients undergoing radiotherapy [84]. Measurement of IL-17 may be useful in assessing patient grading and prognosis, as patients with HGG (including glioma III and IV grade) have significantly elevated levels of IL-17 in blood serum compared to LGG [34].

It appears that in addition to IL-17 itself, IL-17 receptor D (IL-17RD) may be another potential prognostic marker. IL-17RD is a member of the IL-17 receptor family, which regulates cell proliferation, differentiation, survival and inflammation through receptor tyrosine kinase (RTK), fibroblast growth factor (FGF) and IL-17 signaling pathways [85]. To date, the only known ligand of IL-17RD is IL-17A (commonly referred to as IL-17) [85]. There is growing evidence that IL-17RD may play a key role in tumorigenesis the of several malignancies. The role of IL-17RD signaling has been studied in prostate, breast, colorectal, ovarian, and thyroid cancers [85]. Increased expression of IL-17RD in GBM tissue was associated with higher survival rates compared to patients with low IL-17RD expression, while the 3-year OS rates in patients with high IL-17RD were approximately 2,7 fold higher than in the low IL-17RD group (19.5% vs. 7.2%, respectively) [86]. High IL-17RD expression was an independent prognostic factor of favorable prognosis and its evaluation in GBM may help stratify patients’ risk and identify cases requiring more intense treatment and monitoring [86]. In contrast, in Qian et al. study IL-17RD expression was significantly upregulated in GBM tissue and IL-17RD overexpression promoted glioblastoma cells growth and invasion in vitro [87]. To date, only a few studies have investigated the role of IL-17RD in GBM tumorigenesis and prognosis. Since the results have appeared to be contradictory, large-scale cohort studies are necessary to verify the clinical significance of IL-17.

To date, most studies have evaluated IL-17 levels in blood plasma and tumor tissue. Recently, the assessment of IL-17 in cerebrospinal fluid has emerged as another diagnostic modality in brain tumors. The level of IL-6, one of the major Th17-promoting cytokines, was significantly elevated in GBM compared to low-grade gliomas, while a higher level of IL-6 in CSF was associated with high infiltration of tumor-associated macrophages and poor prognosis in patients with GBM [61]. The role of CSF IL-17 as a prognostic biomarker in GBM remains unknown; however, its prognostic and predictive value has been demonstrated in other CNS pathologies. Increased CSF IL-17 was associated with poorer response to initial treatment and relapse in anti-N-methyl-D-aspartate receptor (NMDAR) encephalitis [88]. Furthermore, CSF IL-17 levels correlated positively with the Clinical Assessment Scale in Autoimmune Encephalitis (CASE) score in patients with non-NMDA receptor autoimmune encephalitis. High CSF IL-17 was also associated with intensive care unit admission [89].

In conclusion, IL-17 levels in blood plasma, tumor tissue or CSF may be a potential biomarker in patients with GBM. However, its prognostic value needs to be validated in larger prospective cohort studies. In addition, IL-17 may be a potential biomarker for predicting response to immunotherapy due to its significant impact on anti-tumor immunity. We expect further research in this area in the near future.

## The role of IL-17 and Th17 immunity in the treatment of GBM- current state and future perspectives

Standard therapeutic strategies for GBM, including surgical resection combined with chemotherapy and radiation, are generally ineffective and only modestly prolong patient survival [90]. Recent advances in the understanding of GBM tumorigenesis are providing new insights into potential therapeutic strategies, while novel therapies for patients suffering from GBM are currently emerging. In this section, we will discuss the results of preclinical studies investigating vaccines generating anti-tumor Th17 immunity in the treatment of GBM. We also review the potential anti-cancer agents that exhibit anti-IL-17 activity in glioblastoma. We also provide concise discussion regarding the role of exosome-based immunotherapy and theranostics as promising novel tools in GBM management.

### Anti-cancer vaccines induce Th17 cells and enhance anti-GBM immunity

Anti-cancer vaccines (ACVs) are immunotherapies that work by stimulating the host immune response against tumor-associated antigens. Stimulation of anti-tumor immunity enhances the activity of immune cells to fight cancer cells and provides a durable, long-lasting immune memory that prevents tumor relapse. The major barriers to ACV in glioblastoma are tumor heterogeneity, lack of GBM-specific antigens, antigen loss, a highly immunosuppressive microenvironment, and limited immune cell infiltration into tumor tissue [91]. The efficacy of cancer vaccines in GBM patients has been intensively studied in both preclinical and clinical trials, but the results to date have been underwhelming. [92]. The results of preclinical studies investigating the safety and efficacy of ACV, which acts by stimulating Th17-immunity, are summarized in Table 1.

**Table 1:** Summary of preclinical studies investigating Th17-cells-promoting anti-cancer vaccines in GBM therapy

Vaccine	Biological effects	Study limitations	References
GIFT-7 vaccine	Increased overall survival Generation of durable long-term Th17 cells anti-tumor immune response Irradiation increases vaccine immunogenicity -irradiation decreases the risk of uncontrolled proliferation of vaccine-associated cancer cells Promotion of hyperactive dendritic cells	The anti-tumor effect of the vaccine depends on the age of the murine host Complex process of vaccine construction No data about potential adverse events related to the robust immune response after vaccination In vivo xenograft model (murine preclinical study)	[93]
DC-GL261-ICD vaccine	Induction of anti-GBM immune response (orthotopic model) Vaccine-generated anti-tumor immunity is Th17-depended Expression of Th17 metagene may be associated with better OS in glioma patients Vaccination diminished neurological deficit grade in mice Th17 metagene may be a prognostic factor in GBM	In vitro*, *In vivo xenograft model (subcutaneous and orthotopic) Time and money-consuming process of vaccine construction No data about vaccine toxicity and adverse events	[94]
HSP65-GTL vaccine	Induction of anti-tumor Th17 immunity and expression of IL-17 and IL-21 Recruitment of NK and CD8^+^ cytotoxic cells Increased immune cell infiltration in the GBM milieu	In vivo* orthotopic* murine preclinical study Time and money-consuming process of vaccine construction No data about vaccine-related adverse events	[95]

### Anti-IL-17 can enhance response to immunotherapy

IL-17 appears as one of the key factors in GBM tumorigenesis and resistance to various treatment modalities. IL-17 may drive GBM immunotherapy resistance by maintaining immunosuppression of TME and attenuating anti-tumor immunity. In this paragraph, we discuss potential agents that, due to targeting IL-17, may enhance the efficiency of anti-GBM immunotherapy.

XH30 is a newly described small molecule capable of crossing the BBB. XH30 antitumor activity in GBM was primarily associated with the inhibition of the pro-oncogenic PI3K/AKT signaling pathway [96]. Upregulation of PI3K/AKT signaling in GBM promotes tumor growth, progression, self-renewal, and invasion of nearby tissues. In addition, PI3K/AKT signaling mediates temozolomide resistance [39]. To date, the efficacy of the PI3K inhibitor buparlisib in the treatment of GBM has been investigated in phase I/II clinical trials, but no significant progress has been made [97]. Recently, it has been demonstrated that XH30 administration decreases IL-17 and IL-17R expression and blocks PI3K/AKT signaling in vivo*.* XH30 suppresses GBM growth in subcutaneous and orthotopic mouse models. The authors suggest that the additional anti-GBM effect of XH30, next to the direct PI3K inhibition, is associated with the inhibition of IL-17-mediated activation of the PI3K pathway [37].

Di-amine rosmarinate (FLVM) and imidazole rosmarinate (FLVZ) inhibit IL-17-driven angiogenesis, suppress GBM growth, and increase survival in mice. FLVM also showed an immunomodulatory effect on GBM TME. Both molecules downregulated HIF-1α, reducing hypoxia in the GBM tissue and promoting its apoptosis by increasing the levels of reactive oxygen species [72, 98].

Tumstatin is a novel integrin inhibitor that indirectly suppresses GBM growth by inhibiting angiogenesis in the TME. In addition, tumstatin inhibits IL-17, which promotes GSC viability and GBM growth in vivo in the subcutaneous xenograft mouse model [34, 99].

Temozolomide is the first-line chemotherapeutic agent used to treat GBM. However, the clinical efficacy of temozolomide is frequently limited by the inherent or acquired chemoresistance [100, 101]. Recently, a combination of ulipristal, temozolomide, and hydroxyurea has been demonstrated as a potential approach to increase chemotherapy efficacy in GBM. Combined treatment with three drugs significantly reduced cancer cell proliferation and resulted in the downregulation of GBM-promoting cytokines such as GFβ, IL-10, and IL-17 and the upregulation of GBM-inhibiting IL-23 [65].

Molecule 6, a small molecule characterized by good BBB penetration, high selectivity, and no off-target toxicity, inhibited IL-17-induced GBM cell proliferation via inactivation of IL-17 chaperone proteins. Molecule 6 promoted GBM cell apoptosis, increased oxidative stress leading to cell necrosis, and downregulated VEGF expression in cancer cells. In addition, Molecule 6 exhibited low cytotoxicity [71].

The use of immunotherapy in the treatment of GBM has been studied extensively over the past decades, but no major breakthrough has been achieved [102]. A better understanding of the role of IL-17 in GBM pathogenesis and TME may advance our efforts to effectively combat this malignancy. High Th17 cell commitment in the TME may compromise the antitumor response of host Th1 and CTL cells. Furthermore, the overexpression of Th17 cells in the tumor bed may be the reason for the inefficiency of immunotherapy. Th17 cells induce the exhaustion phenotype of GBM-resident CTL cells, attenuating their antitumor activity [103]. Anti-IL-17 treatment improved response to immunotherapy in gastric and colon cancer cell lines and mouse models of oral cancer [104–106]. The results suggest that combining anti-IL17 agents and immunotherapy is a promising approach to GBM therapy (Table 2); therefore, we expect more research in this area.

**Table 2:** Summary of preclinical studies targeting IL-17 in GBM

Agent	Biological effects	Study limitations	References
XH30	Downregulation of pro-oncogenic PI3K pathway directly and indirectly through inhibition of IL-17 Significant repression of GBM growth in an orthotopic model Downregulation of IL-17R on the surface of cancer cells May increase the anti-GBM efficiency of temozolomide	In vitro and in vivo xenograft murine model No data about XH30 toxicity and adverse events Uncertain mechanism of action	[37, 96]
FLVM, FLVZ	Significant repression of intracranial tumor (orthotopic model) Antiangiogenic activity via IL-17 and VEGF inhibition Potential impact on tumor immune milieu Pro-apoptotic activity No major toxic effects were reported	In vitro and in vivo xenograft murine model Does not fully elucidate the mechanism of action	[72, 98]
Tumstatin	Inhibition of IL-17 promoting effect on subcutaneous GBM tumor (heterotopic model) Repress the stemness effect of IL-17 on glioblastoma cells through impact on transcription factor expression	In vitro and in vivo xenograft murine model (subcutaneous) No data about tumstatin toxicity and observed major adverse events No data about BBB permeability and specificity of tumstatin How tumstatin inhibits IL-17 activity requires deepened investigations	[34, 99]
Ulipristal-temozolomide-hydroxyurea combination	Inhibition of glioblastoma cell proliferation Decrease of total antioxidant capacity, increase of total oxidant status in conditioned medium Downregulation of Th17-related cytokines TGFβ and IL-17	In vitro study No data about cytotoxicity effect on non-cancer cells	[65]
Molecule 6	Low cytotoxic effect on non-cancer cells Inhibition of IL17-induced proliferation of GBM cells Pro-apoptotic activity Promotion of oxidative stress Antiangiogenic effect Good BBB permeability	In vitro study	[71]

### The role of exosome-based immunotherapy and theranostics and their association with IL-17 in GBM

Currently, we notice a rapidly growing interest in exosomes among scientists including cancer researchers. Exosomes are small extracellular vesicles released by various types of cells, which take part in the transmission of biological signals between cells through nucleic acids such as RNA and proteins. Exosomes are present in many extracellular fluids including blood, urine, saliva, and CSF [107]. Exosomes are released by various immune cells including T lymphocytes, NK cells, and macrophages. Exosomes exhibit immunomodulatory activity and regulate the immune response of the host [107].

To date, the potential applications of exosomes have been demonstrated in the diagnosis, prognosis, and treatment of many diseases including brain tumors. In GBM, exosome-based techniques, such as liquid biopsy, offer rapid, repeatable, and non-invasive/less invasive diagnostic methods. Certain tumor cell-derived exosomes may be prognostic biomarkers in GBM [108]. Exosome-based therapeutics also appear as a promising treatment approach in glioblastoma. For example, exosome-based methods may improve drug penetration through BBB and other biological barriers. Furthermore, exosomes exhibit less toxicity and immunogenicity which decreases the risk of adverse events associated with cancer therapy [109]. The role of cell-derived exosomes in GBM immunology and immunotherapy is being intensively studied [109].

Currently, the role of IL-17 and Th17 cells in exosome-based theranostics including diagnosis and treatment remains unknown. However, their applicability has been investigated in other malignancies. In gastric cancer, cancer cells can promote differentiation of Th17 cells through the release of exosome mi-R451 [110]. Overexpression of miR-451 exosome in tumor-infiltrating T cells was associated with more abundant Th17 distribution. In addition, patients with high miR-451 exosome expression in tumor-infiltrating T cells had a significantly worse prognosis. These results suggest that cancer-derived miR-451 exosome may be an unfavorable prognostic biomarker in patients with gastric cancer [110]. The Th17 differentiation-promoting activity of cancer-derived exosomes was also reported in colorectal cancer [111]. Colon cancer-derived exosomal miR-223-3p promoted M2 macrophage polarization resulting in increased secretion of pro-oncogenic IL-17 which intensified the proliferation and migration of colon cancer cells [112]. Breast cancer-derived exosomes through increasing the secretion of proinflammatory cytokines such as IL-17 may enhance the anti-tumor immunity and improve the efficacy of immunotherapy in breast cancer patients [113].

In conclusion, exosome-based theranostics including diagnosis, prognosis, and treatment of glioblastoma is a promising approach that may bypass common obstacles and limitations associated with standard techniques. To date, the association between IL-17 cytokine and exosomes in the context of glioblastoma remains mostly unknown. However, promising results of IL-17 and cell-derived exosomes in other malignancies support the need for further investigations of this issue in glioblastoma.

## Conclusions

The role of IL-17 in GBM tumorigenesis is complex and multidimensional. IL-17 stimulates the PI3K pathway, thereby facilitating GBM cell survival, proliferation and migration. The promotion of GSC survival and self-renewal suggests that IL-17 may be associated with GBM recurrence and radio- and chemoresistance. IL-17 upregulated the expression of the BZW1 oncogene. IL-17 appears to be one of the main drivers of early-stage GBM growth via attenuation of anti-tumor immunity, promotion of survival under hypoxic conditions, and activation of GBM-promoting microglia.

The complex and highly immunosuppressive TME of GBM attenuates the host anti-tumor response. IL-17 facilitates the transmigration of Th17 into the CNS. Th17 can also be generated within the TME. Specific crosstalk between cells in the glioblastoma TME promotes tumor growth, infiltration, and escape from immune surveillance. Given the extensive cytokine network in the GBM niche, targeting a single cell population such as Th17 may not be sufficient to inhibit the tumor-promoting activity of IL-17. IL-17 drives GBM angiogenesis by mediating chronic inflammation and upregulation of VEGF, CD31, and HIF-1α. In addition, high expression of Th17 and VEGF is associated with poorer clinical outcomes in patients with GBM.

The role of IL-17 as a diagnostic and prognostic marker in GBM is ambiguous. The serum concentration of IL-17 may be associated with prognosis in GBM patients undergoing radiotherapy. Moreover, IL-17 levels may predict the clinical course of the disease. Evaluation of IL-17 level may also be useful as a recurrence marker. However, the potential use of IL-17 in clinical practice is associated with several limitations, including its lack of specificity and multifactorial pathogenesis.

Due to its aggressive clinical course and resistance to therapy, GBM is associated with a poor prognosis. There is an urgent need to introduce novel treatment strategies. Considering that IL-17 plays an important role in the pathogenesis of GBM, this cytokine is emerging as a novel therapeutic target in GBM patients. The results of preclinical studies investigating various molecules with anti-IL-17 activity are promising, but require further studies to evaluate their safety and efficacy in humans. Exosome-based immunotherapy and exosome-based theranostics including liquid biopsy are promising novel tools that may overcome common obstacles associated with glioblastoma management. Preclinical studies suggest that vaccine-induced Th17 cells and IL-17 may also have anti-tumor activity in GBM. By generating Th17 immunity, they provide a long-lasting anti-tumor immune response that, if associated with improved prognosis, may soon change the treatment paradigm.

In conclusion, due to its role in tumorigenesis, angiogenesis, and immune surveillance evasion, as well as its potential for aiding in the diagnosis and prognosis of GBM, targeting IL-17 or its signaling pathway shows promise as a novel therapeutic approach for treating GBM. Future research should focus on evaluating the efficacy and safety of anti-cancer molecules with anti-IL-17 activity.

## Data Availability

No datasets were generated or analysed during the current study.

## Declarations

### Research involving human or animal participants

This article does not contain any studies with human participants or animals performed by any of the authors.
